# Sensitivity analysis of rapid antigen tests for the Omicron SARS-CoV-2 variant detection from nasopharyngeal swab samples collected in Santiago of Chile

**DOI:** 10.3389/fpubh.2022.976875

**Published:** 2022-10-20

**Authors:** Carlos Barrera-Avalos, Javier Mena, Roberto Luraschi, Patricio Rojas, Carlos Mateluna-Flores, Eva Vallejos-Vidal, Mónica Imarai, Ana María Sandino, Daniel Valdés, Rodrigo Vera, Iván Hernández, Felipe E. Reyes-López, Claudio Acuña-Castillo

**Affiliations:** ^1^Centro de Biotecnología Acuícola, Facultad de Química y Biología, Universidad de Santiago de Chile, Santiago, Chile; ^2^Departamento de Biología, Facultad de Química y Biología, Universidad de Santiago de Chile, Santiago, Chile; ^3^Hospital de Urgencia Asistencia Pública (HUAP), Santiago, Chile

**Keywords:** rapid antigen test, SARS-CoV-2, Omicron detection, COVID-19, RT-qPCR, detection sensitivity, pandemic in Chile

## Abstract

The COVID-19 pandemic continues to be a concern and keeps global health authorities on alert. The RT-PCR technique has been the gold-standard assay for detecting the SARS-CoV-2 virus. However, rapid antigen tests (RATs) have been widely used to increase the number of tests faster and more efficiently in the population. Nevertheless, the appearance of new viral variants, with genomic mutations associated with greater contagiousness and immune evasion, highlights the need to evaluate the sensitivity of these RATs. This report evaluates the sensitivity of SD Biosensor-Roche, Panbio™, and Clinitest® RATs widely used in Santiago de Chile in the detection of the Omicron variant from Nasopharyngeal samples (NPSs), the most predominant SARS-CoV-2 variant in Chile and the world. SD Biosensor-Roche shows a detection sensitivity of 95.7% in the viral amplification range of 20 ≤ Cq < 25, while Panbio™ and Clinitest® show 100% and 91.3%, respectively. In the viral amplification ranges of 25 ≤ Cq < 30, the detection sensitivity decreased to 28% for SD Biosensor-Roche, 32% for Panbio™, and 72% for Clinitest®. This study indicates that the tested RATs have high sensitivity in detecting the Omicron variant of concern (VOC) at high viral loads. By contrast, its sensitivity decreases at low viral loads. Therefore, it is suggested to limit the use of RATs as an active search method, considering that infections in patients are increasingly associated with lower viral loads of SARS-CoV-2. These antecedents could prevent contagion outbreaks and reduce the underestimation of the current Omicron variant circulation at the local level.

## Introduction

The SARS-CoV-2 virus has kept the medical and scientific community worldwide on constant alert. The appearance of new variants has generated new waves of contagion due to its immune evasion capacity provided by genomic mutations, even in vaccinated patients (1). One of the most valuable tools for controlling the pandemic is the massive and constant population testing, allowing the traceability and timely isolation of infected patients (2). The gold-standard and recommended molecular technique for this testing have been RT-PCR. However, the high demand for this technique, stock shortage of some reagents, and long waiting times for results, has led to the use of alternative detection methodologies, such as rapid antigen tests (RATs). These have been authorized for use in the detection of SARS-CoV-2, associated with a faster and cheaper way of analysis, helping to decongest diagnostic laboratories and increase the testing capacity of the population in different countries. These RATs are based on the detection of viral proteins through antibodies (3). Nevertheless, the constant appearance of SARS-CoV-2 mutations that give rise to different variants could affect its detection performance, making it necessary to evaluate and compare it with the RT-PCR technique. For example, Bayart et al. (4) showed that the detection of some variants such as Delta was 20–40%, while the sensitivity of the Omicron variant was 0–23% for Cq > 25 values in various RATs used in Belgium. Another report indicated a drop of almost 50% sensitivity in RATs in the presence of SARS-CoV-2 mutations associated with the Gamma or Beta variant, or when the viral load decreases (5). In Chile, the most used method for the diagnosis of COVID-19 is the RT-PCR technique, but according to official reports from the Ministry of Health, until March 30, 2022, more than 40% of total the testing tests carried out in Chile corresponded to RATs (6).

SD Biosensor-Roche, Panbio™ and Clinitest® RATs are currently used in Chile, mainly in health centers, and available in pharmacies to detect COVID-19 in symptomatic and asymptomatic patients in a fast way. However, there are no reports of their sensitivities to different viral loads for the Omicron variant of concern (VOC), which is currently predominant in global contagion events (7). This report evaluated three RATs to detect the Omicron variant at different viral loads. Our results show a more than 60% decrease in detection ability against low viral loads for some RATs. This evidence acquires especial relevance considering that currently the Cq values > 25 becoming more predominant in infected patients in Chile.

## Materials and methods

### Clinical samples

Nasopharyngeal swab samples (NPSs) from primary health centers and hospitals belonging to the Central Metropolitan Health Service (CMHS) were used and collected from Dec 27, 2021, to May 21, 2022. The NPSs were taken, preserved, and transported using the CITOSWAB® transport kit with 3 ml of viral transport medium (Cat. No. 2118-0015; Citotest Labware Manufacturing Co., Ltd, Jiangsu, China) to the Virology Laboratory of the Universidad de Santiago de Chile. The NPSs were kept frozen at −80°C after analysis by RT-PCR.

### Rapid antigen tests (RATs)

The SARS-CoV-2 Rapid Antigen Test SD Biosensor-Roche (SD Biosensor inc; South Korea, REF: 9901-NCOV-01G; Lot: 59031G4T1), Panbio™ COVID-19 Ag Rapid Test Device (Abbot; Germany, REF: 41FK10; Lot: 41ADG826A) and CLINITEST® Rapid COVID-19 Antigen Self-Test (Siemens Healthcare; USA, REF GCCOV-502a; Lot: 2001185) RATs were used in this study. All of them were approved for emergency use in Chile and by international health authorities belonging to the International Medical Device Regulators Forum (IMDRF). The RAT SD Biosensor-Roche was used following the manufacturer's instructions from samples in a viral transport medium using 350 μl of NPSs. For Panbio™ and Clinitest® RATs, 350 μl of viral transport medium sample from NPSs were collected and diluted in 300 μl of reaction buffer provided by the manufacturer. The same NPSs were used for the evaluation of the three RATs. All samples were analyzed in the same way. The results were recorded after 15 min.

### COVID-19 diagnosis by RT-PCR and viral load quantification

The detection of viral SARS-CoV-2 was carried out using the ORF1ab probe (TaqMan™ 2019nCoV Assay Kit v1, Thermo Fisher Scientific, Cat. No. A47532) and one-step strategy. Each reaction contained 5 μl of TaqMan™ Fast Virus 1-Step Master Mix 4X, 1 μl of ORF1ab assay 20X, 1 μl of RNase P assay 20X, 11 μl of nuclease-free water, and 2 μl of the extracted RNA sample. The RT-PCR reaction was performed on the Agilent AriaMx Real-Time PCR System (Agilent Technologies, Part. No. G8830A). Serial 1/10 dilutions generated a standard curve with the positive control TaqMan 2019-nCoV Control Kit v1 (10^4^ copies/μL) (Thermo Fisher Scientific, Cat. No. A47533) by RT-PCR. Antilogarithm of the following equation of the line (*y* = −3.07^*^X + 40.2)/2 was used to calculate the viral load. To obtain the number of viral copies per μl, this equation was divided by two according to the volume of RNA (2 μl) used in the RT-PCR reaction. The Cq got in the NPSs was replaced in “*X*”.

### Omicron genotypification by RT-PCR

SARS-CoV-2 positive NPSs were subjected to multiplex RT-PCR for the SARS-CoV-2 variant detection with Allplex™ SARS-CoV-2 Variants I (Cat No. RV10286X) and II (Cat No. RV10305X) Assay Kits, both from Seegene Inc., Seoul, South Korea, following the manufacturer's recommendations. The variant detection kit I detects mutations E484K, N501Y, and HV69/70, while the variant detection kit II detects mutations L452R, W152C, K417T, and K417N. The amplification results were automatically interpreted through the software indicated by the manufacturer.

### Statistical analysis

The statistical relationship between the ratio of Cq > 25 over total Cq value during the three waves of infections in Chile was analyzed by linear regression. Confidence intervals (CI) for proportions set at 95% level of significance were computed from binomial distribution with Wilson method. A value of *p* < 0.05 was observed and considered statistically significant. GraphPrism version 8.0.1 software was used for analysis.

### Ethics statement

This study was authorized by the Ethical Committee of the University of Santiago of Chile (No. 226/2021) and the Scientific Ethical Committee of the Central Metropolitan Health Service, Ministry of Health, Government of Chile (No. 370/2021), and following the Chilean law in force.

## Results

We determine the detection efficiency of three RATs (SD Biosensor-Roche; Panbio™ and Clinites®) authorized for emergency and used massively in Santiago de Chile for the diagnosis of COVID-19. Forty-eight NPSs for SD Biosensor-Roche, Panbio™ and Clinitest® were evaluated. All NPSs were previously diagnosed COVID-19 positive by RT-PCR and characterized for Omicron by genotyping the presence of its characteristic mutations (N501Y/K417N/Δ69-70del, or N501Y/Δ69-70del), as previously indicated for the detection of this variant (8, 9). N501Y/Δ69-70del and N501Y/K417N/Δ69-70del, correspond to subvariants BA.1 and BA.4/5, respectively (8). The circulation frequency of Omicron variants in Chile between December 2021 and May 2022 is shown in Supplementary Figure 1.

**Figure 1:**
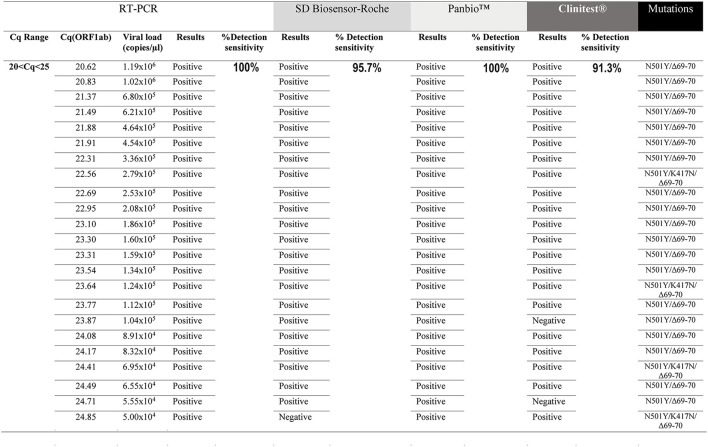
Evaluation of the sensitivity for the rapid antigen test SD Biosensor-Roche, Panbio™ and Clinitest® in detecting the Omicron variant at 20 ≤ Cq < 25 range. All samples were positive for RT-PCR (100%). A total of 23 samples were analyzed. The same NPSs were analyzed by all four methods (RT-PCR and three RATs). N501Y/Δ69-70del and N501Y/K417N/Δ69-70del mutations correspond to subvariants BA.1 and BA.4/5, respectively.

**Figure 2:**
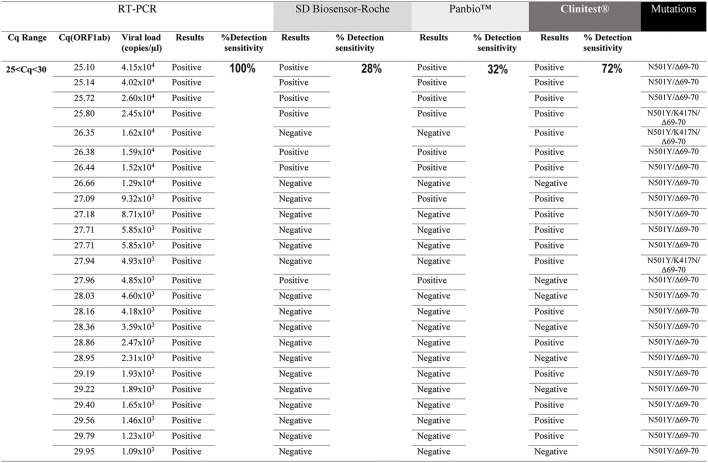
Evaluation of the sensitivity for the rapid antigen test SD Biosensor-Roche, Panbio™ and Clinitest® in detecting the Omicron variant at 25 ≤ Cq < 30 range. All samples were positive for RT-PCR (100%). A total of 25 samples were analyzed. The same NPSs were analyzed by all four methods (RT-PCR and three RATs). N501Y/Δ69-70del and N501Y/K417N/Δ69-70del mutations correspond to subvariants BA.1 and BA.4/5, respectively.

The sensitivity of three rapid antigen tests was tested on RT-PCR positive samples stored in a viral transport medium, between two ranges of Cq amplification values for the viral gene ORF1ab of SARS-CoV-2 (20 ≤ Cq < 25; 25 ≤ Cq < 30). The same NPSs were evaluated by RT-PCR and the three different RATs. The data showed that, under our study conditions, the detection sensitivity for SD Biosensor-Roche in the range of 20 ≤ Cq < 25 was 95.7% (95% CI [79.0–99.2%]). Panbio™ had a detection sensitivity of 100% (95% CI [85.70–100%]) in the same range of Cq (20 ≤ Cq < 25). On the other hand, Clinitest® had a detection sensitivity of 91.3% (95% CI [73.2–97.6%]) (Figure 1). At 25 ≤ Cq < 30, the SD Biosensor-Roche rapid test showed a detection of 28% (95% CI [14.3–47.6%]), the Panbio™ test registered a sensitivity of 32% (95% CI [17.2–51.6%]), while the Clinitest® test showed a detection sensitivity of 72% (95% CI [52.4–87.5%]) (Figure 2). CI values are summarized in Table 1. All samples were positive for RT-PCR in both ranges of Cq values (95% CI [85.7–100%]). We performed a qualitative analysis of the band intensity shown by each RAT in the diagnosis of each NPSs. Interestingly, although the Clinitest® detects SARS-CoV-2 Omicron at low viral loads, the band intensities mainly were lower than the other RATs (Table 2). While, any visible red line is a positive result according to the manufacturer's instructions, denoted as “+” (Supplementary Figure 2). We did the sensitivity analysis but related directly to viral copies/μL. SD Biosensor-Roche detects 100% of samples with 10^6^ and 10^5^ viral copies. However, 10^4^ and 10^3^ viral copies had a sensitivity of 78.6% and 5.9%, respectively (Table 3). On the other hand, Panbio™ detects 100% of 10^6^ and 10^5^ viral copies. Their sensitivity for 10^4^ and 10^3^ viral copies dropped to 85.7% and 11.8%, respectively (Table 3). Clinitest® had a 100% sensitivity to detect Omicron in samples with 10^6^ viral copies. Then, the detection sensitivity was 93.9%, 85.7%, and 64.7% for 10^5^, 10^4^, and 10^3^ viral copies, respectively (Table 3). The Confidence Interval values of each of the viral copy sensitivities are shown in Table 3. These results suggest that the RATs used for diagnosing COVID-19 in Chile have a high sensitivity for sample Cq ranges between 20 ≤ Cq < 25, and for 10^6^, 10^5^, and 10^4^ viral copies, sensitivities close to the minimum recommended for use according to the World Health Organization (WHO) (10). However, its detection capacity decreases against low viral loads of the Omicron variant, between the range of 25 ≤ Cq < 30.

**Table 1:** Precision measurement of the diagnostic sensitivity of the RATs in the two Cq ranges (20 ≤ Cq < 25 and 25 ≤ Cq < 30) with their 95% confidence interval (CI).

**Cq Range**	**RT-PCR**	**SD Biosensor-Roche**	**Panbio™**	**Clinitest®**
20 ≤ Cq < 25	100% [85.7–100%]	95.7% [79.0–99.2%]	100% [85.7–100%]	91.3% [73.2–97.6%]
25 ≤ Cq < 30	100% [86.7–100%]	28.0% [14.3–47.6%]	32.0% [17.2–51.6%]	72.0% [52.4–85.7%]

**Table 2:** Qualitative analysis of the band intensity obtained in the three RATs for the NPSs evaluated in the two Cq ranges.

	**SD Biosensor-Roche**				**Panbio**™				**Clinitest®**			
**Cq range/band intensity**	**–**	**+**	**++**	**+++**	**–**	**+**	**++**	**+++**	**–**	**+**	**++**	**+++**
20 ≤ Cq < 25	1	3	1	18	0	3	2	18	2	0	3	18
25 ≤ Cq < 30	18	3	1	3	17	3	2	3	7	11	6	1

(-): negative (no band); (+): COVID-19 positive, low band intensity; (++) COVID-19 positive, moderate band intensity; (+++): COVID-19 positive, high intensity. Representative images are shown in Supplementary Figure 1.

**Table 3:** Precision measure of diagnostic sensitivity of RATs by viral copies/μl with its 95% confidence interval (CI).

**Viral load (copies/μl)**	**RT-PCR**	**SD Biosensor-Roche**	**Panbio™**	**Clinitest®**
10^6^	100% [34.2–100%]	100% [34.2–100%]	100% [34.2–100%]	100% [34.2–100%]
10^5^	100% [79.6–100%]	100% [79.6–100%]	100% [79.6–100%]	93.9% [70.2–98.8%]
10^4^	100% [78.5–100%]	78.6% [52.4–92.4%]	85.7% [60.1–96%]	85.7% [60.1–96%]
10^3^	100% [81.6–100%]	5.9% [1.0–27%]	11.8% [3.3–34.3%]	64.7% [41.3–82.7%]

**Figure 3:**
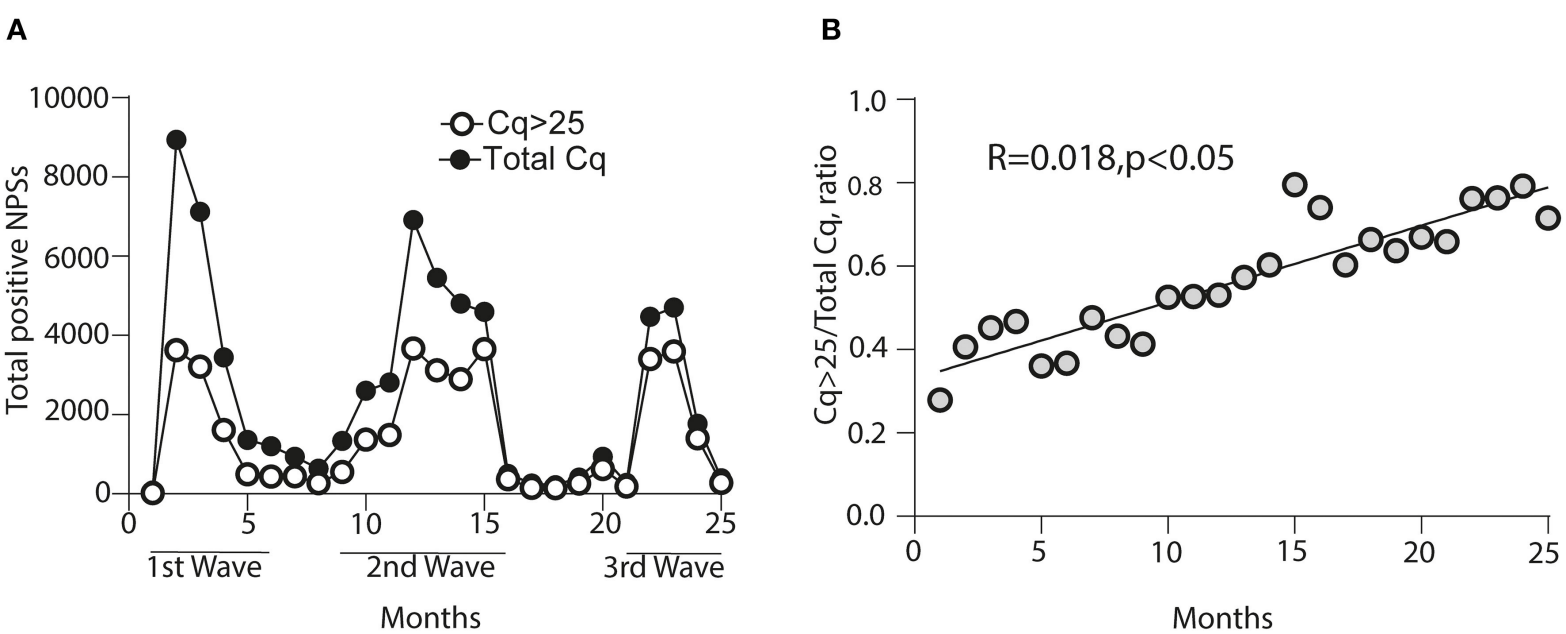
Representation of the proportion for nasopharyngeal swab samples (NPSs) with a Cq-value > 25 through the waves of infections in Chile. **(A)** The first wave corresponds to months 1–6, the second wave to months 9–16, and the third wave to months 21–25 of the pandemic in Chile. Black circles correspond to the total Cq analyzed diagnosed as positive; empty circles represent the samples with a Cq-value > 25 in each wave of infection. **(B)** Linear regression is represented concerning the ratio of Cq-values > 25 over the total Cq analyzed during the first, second, and third waves of infections in Chile.

To determine a possible extent of a lower sensitivity in detecting NPSs following a RAT strategy, we analyzed the proportion of positive samples diagnosed with a Cq value > 25 in Chile's three waves of infections. Thus, we identified an increase in the number of RT-PCR positive for NPSs with Cq values > 25 (open circles) during the pandemic in Chile (Figure 3A). This increase in Cq is statistically significant and exhibits a linear correlation during waves of infection (Figure 3B). It is essential to highlight that the distribution of positive cases with Cq values > 25 represents more than 76% of the total Cq of positive NPSs diagnosed (Table 4). This evidence highlights the special care that must be taken in NPSs with a low viral load diagnosed by RATs.

**Table 4:** Percentage of Cq-values > 25 concerning the total Cq-values in each wave of contagion (1st, 2nd, and 3rd) and months of the pandemic (1–6; 9–16; 21–25) in Chile.

**Waves** **months**	**1st** **(1–6)**	**2nd** **(9–16)**	**3rd** **(21–25)**
Cq > 25	42.50%	59.0%	76.0%

## Discussion

Several works have documented the detection sensitivities of different rapid antigen tests to detect SARS-CoV-2 variants in different ranges of Cq. For example, Osterman et al. reported among all the RATs analyzed that the FUJIFILM COVID-19 Ag Test has a 31.4% detection of the Omicron variant in the range of Cq < 25. Interestingly, a 0% detection sensitivity was reported for amplification values of Cq > 25 (11). The Medicovid-AG SARS-CoV-2 Antigen Rapid Test Card-nasal test had almost 80% detection in the ranges of Cq < 25, although its detection sensitivity decreased to 8.3% in Cq > 25 for the same Omicron variant (11). Bayart et al. (4) reported that RATs used in Belgium, such as the Sejoy SARS-CoV-2 Antigen Rapid Test and New-gene COVID-19 Antigen Detection Kit, have 97% detection sensitivity for the Omicron variant in ranges of Cq < 25. However, in Cq > 25 decrease to 7.7%. The RAT Ag 2019-nCoV-PROGNOSIS, used in the city of Larissa, Greece, in the Omicron wave, showed a reduction of almost 40% in its sensitivity compared to the Alpha variant (12). Other studies have determined that RATs such as SD Biosensor-Roche, decrease their sensitivity from 91% to 42% against samples with mutations related to Gamma or Beta, even in ranges with high viral loads (20 ≤ Cq < 25). The sensitivity dropped to 0% when detecting samples with the same mutations in the range of 25 < Cq < 30 (5). These investigations indicate that RATs have high efficiency in detecting high viral loads (Cq < 25), even in the presence of different variants. By contrast, RATs reduce their sensitivity at low viral loads (ranges of Cq > 25), even in the presence of SARS-CoV-2 variants such as Omicron, the predominant in the world today according to a WHO statement (7). To date, no report has estimated the sensitivity of the RATs used for the diagnosis of COVID-19 associated with Omicron in Chile. Furthermore, none of the above information analyzes the increase in Cq during the pandemic in the countries of origin. This evidence is important because values of Cq > 25 would promote a more significant number of false negatives using the different RATs compared to previous waves of contagion, according to the evidence of sensitivity described above. In this study, the sensitivity of three RATs used for diagnosing COVID-19 in Chile was evaluated and compared with the RT-PCR technique. The data under our study conditions, indicated that for high viral loads (20 ≤ Cq < 25), the SD Biosensor-Roche, Panbio™ and Clinitest® tests have 98.7%, 100%, and 91.3% detection, which decreases to 28%, 32%, and 72% for samples with 25 ≤ Cq < 30, respectively. This could have an epidemiological incidence. According to the official report of the Chilean Ministry of Health, at March 30, 2022, during the third wave by Omicron infections, 69,702 tests were carried out. Of them, 44% were RATs and 66% by RT-PCR, yielding 5,603 positive tests (6). This could mean that of the 5,603 positive NPSs, about 2,400 NPSs were diagnosed by RATs. Of this 2,400 positive NPSs, ~1,800 samples had a Cq > 25 (76% of the positive samples in the third wave). Therefore, this could mean that the 1,800 samples positive for RATs could represent 28% detection if, for example, RAT SD Biosensor-Roche had been used, giving ~4,600 samples that could have been potentially reported as false negatives.

In our study, we genotyped the Omicron variant for either three (N501Y/K417N/Δ69-70del) or two (N501Y/Δ69-70 del) mutations of the spike (S) protein. The RT-PCR does not detect the S target gene because this variant contains the deletion at position 69-70 (Δ69-70del), termed S gene target failure (SGTF), according to the Centers for Disease Control and Prevention (CDC) (13). Although this deletion is also found in the alpha variant (14), in Chile, the alpha variant is considered as not circulating in the year 2022, while the Omicron Variant represents 100% of the predominant variant since February 2022 (15). The other mutations (N501Y and K417N) are also used for Omicron genotyping and previously reported in other countries and subvariants (8, 9). Due to the large number of mutations found in the S protein, most RATs detect the N gene (Nucleocapsid) of SARS-CoV-2 due to its higher conservation rate (16) including the rapid tests analyzed in this work [Revised in (17)]. However, mutations also affect this gene. For example, a study by Jian et al. (18) determined that the Panbio™ nasopharyngeal test decreases its sensitivity for detecting the Alpha variant due to the T135I mutation in the N gene. Although Omicron also has P13L, Δ31–33, R203K, and G204R mutations in the nucleocapsid, other variants like Delta have D63G, R203M, and D377Y mutations (19–21). These differences in mutations could account for the decreased sensitivity of RATs against the Omicron variant. The results presented in this report for Panbio™ RAT do not agree with the report of de Michelena et al. made in Spain. They used only samples from patients with symptoms, indicating a sensitivity of 87.2% for Omicron samples in those with amplification values of Cq < 30, and an 82.8% sensitivity for samples Cq < 35. Interestingly, for samples with Cq < 25 they reported 92.5% sensitivity, similar to our data (22). The results of Deerain et al. (23) indicated that both the SD Biosensor-Roche and Panbio™ tests did not detect either the Delta or Omicron variant in Cq ranges close to 28, like the results presented in our study. It is difficult to compare the viral loads for the different Cq cut-offs considered in the other investigations since it depends on the specific chemical and parameters established for each RT-PCR kit. According to the Food and Drug Administration, the best way to evaluate the performance of the other RATs is by using an active virus (24). In line with this idea, a report using an active virus cultured *in vitro* in Vero cells indicated a sensitivity of 67% and 36% for detecting Delta and Omicron, respectively (25).

The effect observed in the decrease of viral loads (higher Cq-values) in Chile may be due to the massive vaccination of the population. To date, it reaches more than 91% population with a complete schedule (26). This may suggest that in countries with high vaccination rates, the effect could be the same, thus increasing the number of false negatives in using RATs with lower sensitivity. Therefore, this study suggests limiting the use of some RATs in the current wave of Omicron in Chile and the rest of the world because they do not meet the minimum sensitivity criteria of 80%, according to the WHO (10). Contrary to the high percentage of detection that we report for Clinitest® in low viral loads (Cq > 25), other reports have reported 0% detection for the same range of values, even in Omicron-viral loads of the order of 10^5^, where according to our data, Clinitest® had almost 94% detection. These differences may be due to the additional initial treatment of the NPSs performed by Bayart et al. (4). In fact, and according to our data, Clinitest® had almost 94% detection. Regardless of the detection of the Omicron variant, our results are similar to those reported by Merino-Amador et al. (27), who indicated a sensitivity of 98% in samples Cq < 25 and 80% in NPSs Cq>25, without prior maintenance or dilution with viral transport medium. Our results are almost similar to the Siemens Healthineers manufacturer, which reports a 97% detection rate for Omicron. However, the ranges of Cq analyzed were not indicated (28).

Notably, the Panbio™ and Clinitest® RATs do not have instructions for evaluating NPSs contained in viral transport medium. Therefore, the observed results, although consistent with what is reported in the literature, reflect a dilution of the NPSs. This can shift Cq values up to 3 cycles. For example, for the Panbio™ RAT, the manufacturers claim a sensitivity of 99% Cq ≤ 33, therefore, they recommend not using Cq > 29 samples when they are contained in a viral transport medium (29). Although validation studies of Panbio™ using NPS in transport buffer were reported, the results indicated an overall sensitivity of 72.6%, without showing the variant of SARS-CoV-2 used (30), in any case, the results shown for Panbio™ and Clinitest® are higher to those demonstrated by SD Biosensor-Roche, who, despite having instructions to measure NPSs in viral transport medium and sample dilution, had the lowest performance of the analysis. Importantly, our results were consistent even though in our evaluation of the Panbio™ and Clinitest® RATs we did a dilution (NPSs maintained in viral transport medium) prior to the analysis of the sample. Accordingly, de Michelena et al. (22) reported a sensitivity of 92.6% in Cq < 25 for Omicron in undiluted NPSs in viral transport medium. While in some cases, we had better results in the sensitivity of Clinitest®, even when we conducted the analysis from a viral transport medium, where some reports indicated up to 0% sensitivity to detect Omicron at Cq > 25 (4). An important point to consider is that the NPSs were kept at −80°C immediately after their analysis by RT-PCR. These were thawed for evaluation by the different RATs. While this may affect the integrity of the SARS-CoV-2 N protein, one thaw cycle is unlikely to affect the overall result of our study. Our results suggest that the sensitivity of the RATs decreases concerning the viral load and not to the Omicron variant because, at high viral loads, detection even reaches 100%.

This study is the first report that analyzes some of the main RATs used in the public health system in Chile to detect Omicron. Taking into consideration our results come from NPSs, it is necessary to evaluate the RATs from manufacturers that use saliva samples, which have also been widely used, especially in hospitalized patients (31), where their sensitivity is even lower compared to NPSs (32). In Chile, public health policies establish that performing RT-PCR is mandatory in case of negative results obtained from RAT. However, it is unclear the number of patients involved in this procedure and whether this protocol is effectively applied to all NPSs. Likewise, it is recommended that after 2 years of the COVID-19 pandemic, health organizations analyze the efficiency and sensitivity of RATs since viral loads are increasingly low, and their high sensitivity for Cq < 25 could decrease, especially against new viral variants. Taking together, the results obtained for the three RATs assessed under our experimental conditions provide helpful information for possible decision-making in public health policies at the local and global level.

## Data availability statement

The original contributions presented in the study are included in the article/Supplementary material, further inquiries can be directed to the corresponding authors.

## Ethics statement

The studies involving human participants were reviewed and approved by Ethical Committee of the University of Santiago of Chile (No. 226/2021) and the Scientific Ethical Committee of the Central Metropolitan Health Service, Ministry of Health, Government of Chile (No. 370/2021). Written informed consent for participation was not required for this study in accordance with the national legislation and the institutional requirements.

## Author contributions

CA-C and FER-L: conceptualization. CB-A: methodology and formal analysis. FER-L and MI: validation. JM, RL, RV, CM-F, and IH: investigation. CA-C, MI, FER-L, and AMS: resources. CA-C and CB-A: data curation and writing—original draft preparation. CA-C, FER-L, CB-A, and EV-V: writing—review and editing. DV: visualization. CA-C, FER-L, and AMS: supervision. FER-L and AMS: project administration and funding acquisition. All authors have read and agreed to the published version of the manuscript.

## Funding

The Laboratory of Virology had the support from the COVID-19 diagnosis in the University laboratories network (Ministry of Sciences, Ministry of Health, Government of Chile) for diagnosis tasks. The authors also thank to the rapid assignment of resources for research projects on the Coronavirus pandemic (COVID-19) (project number COVID1038; ANID, Government of Chile), Fondecyt regular project numbers 1201664 (MI) and 1211841 (FER-L), and Fondecyt iniciación grant 11221308 (ANID, Government of Chile). DICYT-USACH project number 021943AC (CA-C) grants. The funders had no role in study design, data collection, and analysis, decision to publish, or preparation of the manuscript.

## Conflict of interest

The authors declare that the research was conducted in the absence of any commercial or financial relationships that could be construed as a potential conflict of interest.

## Publisher's note

All claims expressed in this article are solely those of the authors and do not necessarily represent those of their affiliated organizations, or those of the publisher, the editors and the reviewers. Any product that may be evaluated in this article, or claim that may be made by its manufacturer, is not guaranteed or endorsed by the publisher.
