# Supplementary material for: Sensitivity analysis of rapid antigen tests for the Omicron SARS-CoV-2 variant detection from nasopharyngeal swab samples collected in Santiago of Chile
Source: Front Public Health. 2022 Oct 20;10:976875. doi: 10.3389/fpubh.2022.976875 (PMC9631301; doi:10.3389/fpubh.2022.976875)
Supplement: Supplementary file 1 [file Data_Sheet_1.pdf]

SUPPLEMENTARY MATERIAL

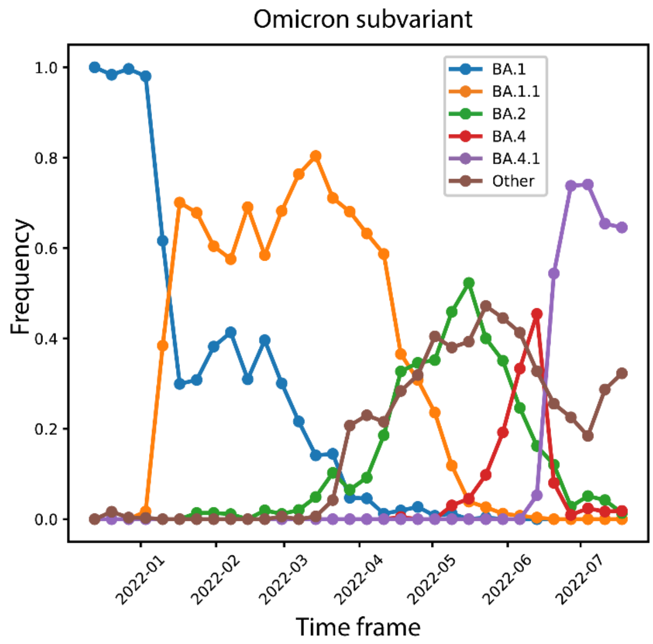

Supplementary Figure 1. Circulation of Omicron Subvariants in Chile. The circulation frequency of the different Omicron subvariants of SARS-CoV-2 is shown between December 2021 and May 2022 (sampling collection time for the NPSs assessed). Different colors denote a particular subvariant. Data obtained from the public database of the Ministry of Health, Government of Chile (https://vigilancia.ispch.gob.cl/app/varcovid)

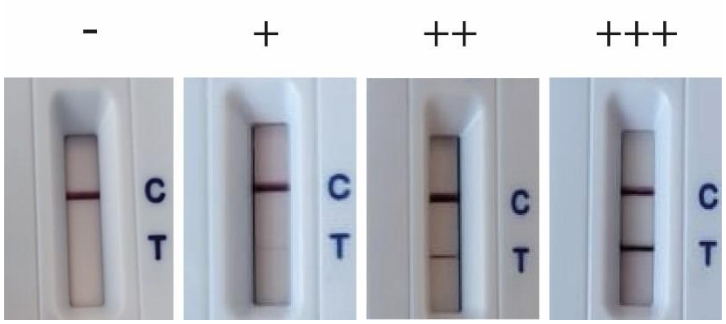

Supplementary Figure 2. Representative images for the qualitative analysis of the band intensity obtained in the three RATs for the NPSs evaluated in the two Cq ranges. A symbol was assigned according to the band intensity. (-): negative (no band); (+): COVID-19 positive, low band intensity; (++) COVID-19 positive, moderate band intensity; (+++): COVID-19 positive, high intensity.
